# Phenolic Acid Derivatives, Flavonoids and Other Bioactive Compounds from the Leaves of *Cardiocrinum cordatum* (Thunb.) Makino (Liliaceae)

**DOI:** 10.3390/plants10020320

**Published:** 2021-02-07

**Authors:** Kengo Hori, Takashi Watanabe, Hari Prasad Devkota

**Affiliations:** 1 Graduate School of Pharmaceutical Sciences, Kumamoto University, 5-1 Oe-honmachi, Chuo-ku, Kumamoto 862-0973, Japan; 209y3008@st.kumamoto-u.ac.jp (K.H.); wtakashi@kumamoto-u.ac.jp (T.W.); 2 Analytical Research Laboratories, Pharmaceutical Technology, Astellas Pharma Inc., 160-2, Akahama, Takahagi-shi, Ibaraki 318-0001, Japan; 3 Program for Leading Graduate Schools, Health Life Science: Interdisciplinary and Glocal Oriented (HIGO) Program, 5-1 Oe-honmachi, Chuo ku, Kumamoto 862-0973, Japan

**Keywords:** *Cardiocrinum cordatum*, Ubayuri, phenylpropanoids, flavonoid, caffeic acid, chemotaxonomy

## Abstract

*Cardiocrinum cordatum* (Thunb.) Makino (Family: Liliaceae), commonly known as ‘Ubayuri’, is native to Japan and some islands in the Russian Far East. It has high value as food, medicinal, and ornamental species. The aim of this study was to isolate and characterize the main chemical constituents of the leaves of *C. cordatum*. A total of 19 compounds, namely caffeic acid (**1**), caffeic acid methyl ester (**2**), caffeic acid *β*-glucopyranosyl ester (**3**), caffeic acid 4-*O*-*β*-glucopyranoside (**4**), ferulic acid (**5**), isoferulic acid (**6**), protocatechuic acid (**7**), syringic acid (**8**), 2,6-dimethoxy-*p*-hydroquinone 1-*O*-*β*-glucopyranoside (**9**), esculetin (**10**), taxifolin (**11**), quercetin 3-*O*-(6-*O*-*α*-rhamnopyranosyl)*β*-glucopyranoside-7-*O*-*β*-rhamnopyranoside (**12**), 2,7-dimethyl-2,4-diene-deca-*α*,*ω*-diacid *β*-glucopyranoside (**13**), 4-[formyl-5-(methoxymethyl)-1*H*-pyrrol-1-yl]butanoic acid (**14**), (3*Z*)-3-hexenyl *β*-glucopyranoside (**15**), tryptophan (**16**), adenine (**17**), adenosine (**18**), and 2-deoxyadenosine (**19**) were isolated using various chromatographic methods. The structures of isolated compounds were elucidated on the basis of their NMR spectroscopic data. All these compounds were isolated for the first time from the genus *Cardiocrinum*. Phenolic acid derivatives and flavonoids can be considered as chemotaxonomic markers in the leaves of *Cardiocrinum* species.

## 1. Introduction

**Figure 1:**
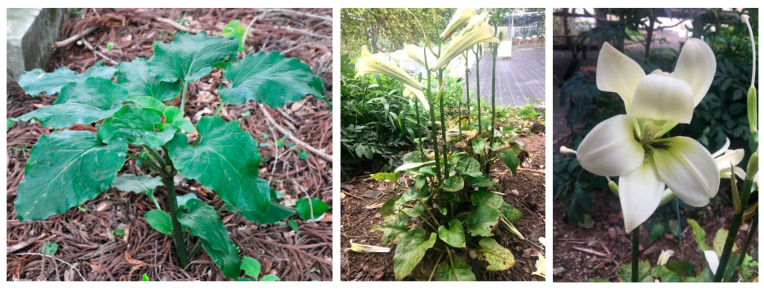
Photographs of *Cardiocrinum cordatum* (From left: Young leaves, plant at flowering stage and flower).

The genus *Cardiocrinum* (Liliaceae) comprises of three perennial bulbiferous herb species, *C. cathayanum* (E. H. Wilson) Stearn, *C. cordatum* (Thunb.) Makino., and *C. giganteum* (Wall.) Makino [1,2]. Among them, *C. cathayanum* is endemic to China [1,3] and *C. giganteum* is distributed in Bhutan, China, India, Myanmar, and Nepal [1,3,4]. *Cardiocrinum cordatum* (Figure 1), commonly known as ‘Ubayuri’ in Japan, is native to Japan and some islands in the Russian Far East [1,5]. It is a perennial herb with the height of about 60–100 m. In traditional medicines, the bulbs are used for the treatment of fever and stomachache [6]. The starch obtained from the bulbs is used as food [6,7,8]. Although the *Cardiocrinum* species have high value as food, medicinal, and ornamental plants [4,6], only a few studies have been performed regarding their chemical constituents. Some previous studies reported the flavonoids from the seeds of *C. giganteum* [9,10,11] and the starch composition of the bulbs of *C. cordatum* var. *glehnii* (F.Schmidt) H.Hara [7,8]. However, there is no report on the chemical constituents of the leaves of *C. cordatum*. Detailed understanding of bioactive chemical composition of plant species is necessary to explore their potential as raw materials for pharmaceutical, cosmetic, and nutraceutical also to understand the role of these compounds in chemotaxonomy. Thus, the main aim of this study was to isolate and identify the major chemical constituents of the leaves of *C. cordatum*.

## 2. Results and Discussion

**Figure 2:**
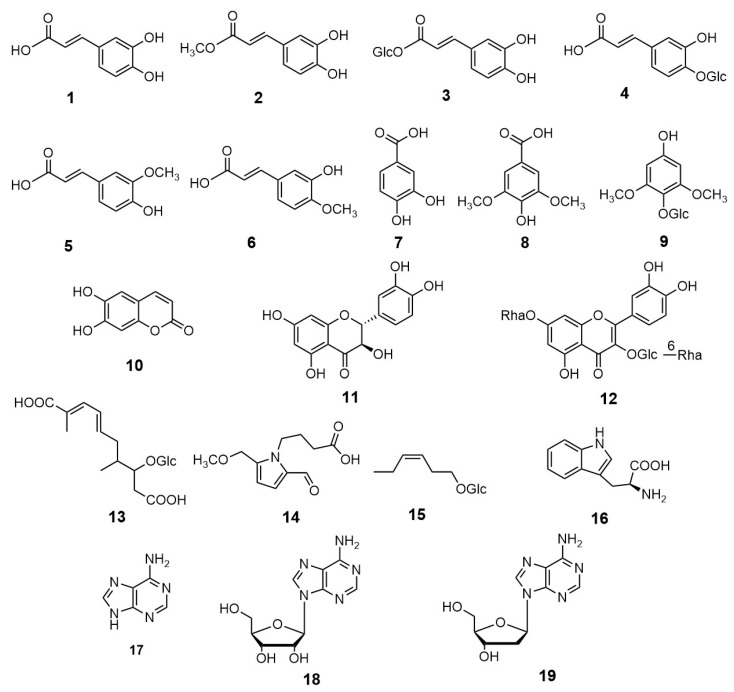
Structures of compounds isolated from *Cardiocrinum cordatum.*

The dried young leaves of *C. cordatum* were extracted with methanol (MeOH) and the extract was then subjected to repeated column chromatography on MCI gel CHP20P, Sephadex LH-20, ODS and silica gel to isolate 19 pure compounds. The structures of isolated compounds were elucidated on the basis of their ^1^H, ^13^C- and 2D-nuclear magnetic resonance (NMR) spectroscopic data, and by comparison with literature values as caffeic acid (**1**) [12], caffeic acid methyl ester (**2**) [12], caffeic acid *β*-glucopyranosyl ester (**3**) [13], caffeic acid 4-*O*-*β*-glucopyranoside (**4**) [13], ferulic acid (**5**) [14], isoferulic acid (**6**) [14], protocatechuic acid (**7**) [15], syringic acid (**8**) [15], 2,6-dimethoxy-*p*-hydroquinone 1-*O-β*-glucopyranoside (**9**) [16], esculetin (**10**) [17], taxifolin (**11**), quercetin 3-*O*-(6-*O*-*α*-rhamnopyranosyl)*β*-glucopyranoside-7-*O*-*α*-rhamnopyranoside (**12**) [18], 2,7-dimethyl-2,4-diene-deca-*α*,*ω*-diacid *β*-glucopyranoside (**13**) [19], 4-[formyl-5-(methoxymethyl)-1*H*-pyrrol-1-yl]butanoic acid (**14**) [20], (3*Z*)-3-hexenyl *β*-glucopyranoside (**15**) [21], tryptophan (**16**), adenine (**17**), adenosine (**18**) [22] and 2-deoxyadenosine (**19**) [22] (Figure 2).

All these compounds belonging to different chemical classes such as phenylpropanoids (**1**–**6**), simple phenolic compounds (**7**–**9**), coumarin (**10**) and flavonoids (**11**, **12**), 2,7-dimethyl-2,4-diene-deca-*α*,*ω*-diacid glucopyranoside (**13**), 4-[formyl-5-(methoxymethyl)-1*H*-pyrrol-1-yl] butanoic acid (**14**), (3*Z*)-3-hexenyl *β*-glucopyranoside (**15**), amino acid (**16**), and nucleic acid derivatives (**17**–**19**) were isolated and identified for the first time from the genus *Cardiocrinum*. The genus *Cardiocrinum* comprises only three species, *C. cathayanum*, *C. cordatum,* and *C. giganteum* [1,2]. Further, two varieties of *C. giganteum* i.e., *C. giganteum* var. *giganteum* and *C. giganteum* var. *yunnanense* (Leichtlin ex Elwes) Stearn [3], and one variety of *C. cordatum*, i.e., *C. cordatum* var. *glehnii* (F.Schmidt) H.Hara. are reported [8]. *Cardiocrinum* species are not well explored regarding their chemical constituents except the isolation and identification of bioflavonoids from the seeds of *C. giganteum*. Shou et al. [10] reported two new racemic bioflavonoids, CGY-1 ((*S*)-2*R*,3″*R*- and (*R*)-2″S,3″*S*-dihydro-3″-hydroxyamentoflavone-7-methyl ether) and CGY-2 ((*S*)-2″*R*,3″*R*- and (*R*)-2″*S*,3″*S*-dihydro-3″-hydroxyamentoflavone) from the 95% ethanol extract of the seeds of *C. giganteum* var. *giganteum* having antitussive properties. CGY-1 was further reported as a potent agent to improve memory deficit for the treatment of cognitive dysfunction [9]. Similarly, Xia et al. [11] reported the isolation of a new biflavonoid, 3”-hydroxyrobustaflavone along with 3”-hydroxyamentoflavone, quercetin, apigenin, and kaempferol with antioxidative activities from the 95% ethanol extract of seeds of *C. giganteum* var. *yunnanense*.

Phenolic acids belonging to the both groups of hydroxycinnamic acid derivatives (phenylpropanoid derivatives) and hydroxybenzoic derivatives are abundantly present in many plant species and specially in berries, fruits, and vegetables [23,24]. Phenolic acids and flavonoids are reported to play an important role in plants as antioxidants, as defense regulators, during unfavorable conditions such as drought and infections and as signaling molecules in plant-microbe symbiosis [25,26,27,28,29,30,31]. These compounds exert potent pharmacological activities in humans and animals and are of great interest as food, nutrition, and medicine [32,33,34,35,36]. Phenolic acid derivatives and flavonoids are reported as common constituents of plants of various genera of Liliaceae family such as *Fritillaria* [37], *Hosta* [38], and *Lilium* [39]. Another compound, 2,7-dimethyl-2,4-diene-deca-*α*,*ω*-diacid *β*-glucopyranoside (**13**) was reported previously from *Orycatanthus* sp. (Loranthaceae) [19] and *Cydonia vulgaris* Pers. (Rosaceae) [40]. It was reported as a potent antitumor compound as it inhibited the ligand binding to vascular endothelial growth factor (VEGF) receptor [19]. Its aglycone, 2,7-dimethyl-2,4-diene-deca-*α*,*ω* -diacid, was reported from *Phaseolus multiflorus* Lam. (Fabaceae) [41]. Similarly, 4-[formyl-5-(methoxymethyl)-1*H*-pyrrol-1-yl]butanoic acid (**14**), a pyrrole alkaloid derivative, was previously reported from *Lycium chinense* Mill. (Solanaceae) as a hepatoprotective compound [42]. (3*Z*)-3-Hexenyl *β*-glucopyranoside (**15**) has been previously reported from *Pertya glabrescens* Sch.Bip. (Asteraceaae) [43], Codonopsis Radix [21], *Celosia argentea* L. (Amaranthaceae) [44], *Roscoea purpurea* Sm. (Zingiberaceae) [45], among others. An amino acid, tryptophan (**16**) and three nucleic acid derivatives (**17**–**19**), which are common primary metabolites, were also isolated and identified in this study.

As there have been no previous studies on the leaves of other *Cardiocrinum* species and varieties, a chemotaxonomic comparison at present is difficult and needs further research. These compounds isolated in this study might also be specific to one genotype of the plant species under investigation in this study, thus the comparison of the chemical constituent profile of plants collected in different localities in necessary. As only very few studies have reported the activity of isolated compounds form *Cardiocrinum* plants, thus detailed bioassay guided isolation of the compound may result in the isolation of other bioactive compounds. Similarly, the quantitative analysis of the compounds may also help to explore these species as potential new sources of bioactive compounds.

## 3. Materials and Methods

### 3.1. General Experimental Procedures

^1^H-, ^13^C- and 2D-NMR spectra were measured on Bruker AVANCE-I 600 NMR Spectrometer (^1^H-NMR: 600 MHz and ^13^C-NMR: 125 MHz). Column chromatography (CC) was carried out with MCI gel CHP20P (75–150 μm, Mitsubishi Chemical Industries Co., Ltd., Tokyo, Japan), Sephadex LH-20 (Amersham Pharmacia Biotech, Tokyo, Japan) and Chromatorex ODS (30–50 μm, Fuji Silysia Chemical Co., Ltd., Aichi, Japan). TLC was performed on a precoated silica gel 60 F254 (0.2 mm, aluminum sheet, Merck KGaA, Darmstadt, Germany).

### 3.2. Plant Materials

The young leaves of *C. cordatum* were collected in Kochi Prefecture, Japan in May 2015 and identified by Prof. Takashi Watanabe, Kumamoto University. A voucher specimen (No. KUT [exp]004) has been deposited at the Department of Medicinal Botany, School of Pharmacy, Kumamoto University, Kumamoto, Japan.

### 3.3. Extraction and Isolation

The shade dried young leaves of *C. cordatum* (960 g) were extracted twice with MeOH (15 L each time). The extracts were then combined and evaporated under reduced pressure to give 202.0 g extract. The extract was then subjected on MCI gel CHP20P column chromatography (CC) and eluted successively with water, 20%, 40%, 60%, 80%, 100% MeOH and CHCl_3_:MeOH (1:1) to give 10 fractions (1–10). Fraction 3 (5.8 g, H_2_O eluate) was subjected on Sephadex LH-20 CC (50% MeOH) to give four subfractions (3-1–3-4). Fraction 3-4 was subjected on ODS CC (5–100% MeOH) to afford compound **7** (9.7 mg). Fraction 4 (3.0 g, H_2_O eluate) was subjected on Sephadex LH-20 CC (50% MeOH) to give seven subfractions (4-1–4-7). Subfraction 4-3 (311.0 mg) was subjected to repeated column chromatography on silica gel CC (CH_2_Cl_2_:MeOH:H_2_O = 8:2:0.1) to afford compound **9** (6.9 mg). Subfraction 4-4 (589.0 mg) was subjected to silica gel CC (CH_2_Cl_2_:MeOH:H_2_O = 8:2:0.1) followed by ODS CC (10% MeOH) to afford compounds **17** (6.1 mg) and **18** (1.4 mg) and **19** (4.2 mg). Subfraction 4-6 (138.3 mg) was ODS CC (20–40% MeOH) followed by repeated CC using Sephadex LH-20 (50% MeOH) to afford compounds **1** (4.7 mg), **3** (14.2 mg), **4** (11.0 mg), and **16** (23.0 mg). Fraction 5 (711.0 mg, 20% MeOH eluate) was subjected to Sephadex LH-20 CC (50% MeOH) and then to repeated CC on silica gel (CH_2_Cl_2_:MeOH:H_2_O = 8:2:0.1) to afford compound **10** (2.4 mg). Fraction 6 (3.98 g, 40% MeOH eluate) was subjected to Sephadex LH-20 CC (50% MeOH) to obtain 4 subfractions (6-1–6-4). These subfractions were further purified using silica gel, Sephadex LH-20, and ODS CC to afford compounds **12** (15.9 mg), **13** (4.3 mg), **14** (11.0 mg) and **15** (4.6 mg). Fraction 7 (4.8 g, 60% MeOH eluate) was subjected to silica gel CC (CH_2_Cl_2_:MeOH:H_2_O = 8:2:0.1) to obtain 5 subfractions (7-1–6-5). These subfractions were further purified using silica gel, Sephadex LH-20 and ODS CC to afford compounds **2** (1.8 mg), **5** (4.1 mg), **6** (1.1 mg), **8** (5.0 mg), and **11** (11.5 mg).

### 3.4. Esculetin *(**10**)*

^1^H-NMR (in CD_3_OD) *δ*_H_ 7.84 (1H, d, *J* = 9.4 Hz, H-4), 7.34 (1H, s, H-8), 7.04 (1H, s, H-7), 6.21 (1H, d, *J* = 9.4 Hz, H-3). ^13^C-NMR (in CD_3_OD) *δ*_C_ 163.7 (C-2), 153.4 (C-7), 152.7 (C-6), 146.1 (C-4), 144.5 (C-9), 116.8 (C-5), 113.2 (C-3), 112.9 (C-10), 104.4 (C-8).

### 3.5. 2,7-Dimethyl-2,4-diene-deca-α,ω-diacid β-glucopyranoside *(**13**)*

^1^H-NMR (in CD_3_OD) *δ*_H_ 7.17 (1H, d, *J* = 11.4 Hz, H-3), 6.47 (1H, dd, *J* = 14.9, 11.4 Hz, H-4), 6.11 (1H, dt, *J* = 14.9 Hz, H-5), 4.33 (1H, d, *J* = 7.6 Hz, Glc-1), 4.02 (1H, m, H-8), 3.83–3.65 (2H, m, Glc-6), 3.45–3.15 (4H, m, Glc-2–Glc-5), 2.64–2.54 (2H, m, H-9), 2.47–2.16 (2H, m, H-6), 1.95 (1H, m, H-7), 0.95 (3H, d, *J* = 6.8 Hz, H-11). ^13^C-NMR (in CD_3_OD) *δ*_C_ 176.6 (C-10), 172.3 (C-1), 142.6 (C-5), 140.1 (C-3), 129.0 (C-4), 126.5 (C-2), 104.8 (Glc-1), 81.9 (C-8), 78.2 (Glc-3), 77.8 (Glc-5), 75.4 (Glc-2), 71.6 (Glc-4), 62.9 (Glc-6), 39.4 (C-9), 38.7 (C-7), 37.0 (C-6), 15.6 (C-11), 12.7 (C-12).

### 3.6. 4-[Formyl-5-(methoxymethyl)-1H-pyrrol-1-ly]butanoic acid *(**14**)*

^1^H-NMR (in CD_3_OD) *δ*_H_ 9.45 (1H, s, CHO), 6.98 (1H, d, *J* = 4.0 Hz, H-3), 6.28 (1H, d, *J* = 4.0 Hz, H-4), 4.49 (2H, s, H-6), 4.37 (2H, t, *J* = 7.3 Hz, H-1′), 3.35 (3H, s, OCH_3_), 2.32 (2H, t, *J* = 7.3 Hz, H-3′), 2.01 (2H, q, *J* = 7.3 Hz, H-2′). ^13^C-NMR (in CD_3_OD) *δ*_C_ 181.1 (CHO), 176.6 (COOH), 141.1 (C-5), 133.9 (C-2), 125.9 (C-3), 112.9 (C-4), 66.4 (C-6), 58.3 (OCH_3_), 45.9 (C-1′), 31.8 (C-3′), 27.7 (C-2′).

### 3.7. (3-Z)-3-Hexenyl β-glucopyranoside *(**15**)*

^1^H-NMR (in CD_3_OD) *δ*_H_ 5.47 (1H, dt, *J* = 10.7 Hz, H-3), 5.37 (1H, dt, *J* = 10.7, 7.0 Hz, H-4), 4.33 (1H, d, *J* = 7.5 Hz, Glc-1), 3.86 (2H, m, Glc-6), 3.68–3.18 (4H, m, Glc-2–Glc-5), 2.37 (2H, q, H-2), 2.05 (2H, q, H-5), 0.94 (3H, t, H-6); ^13^C-NMR (in CD_3_OD) *δ*_C_ 135.3 (C-3), 125.8 (C-4), 104.1 (Glc-1), 77.7 (Glc-3,Glc-5), 74.9 (Glc-2), 71.4 (Glc-4), 71.0 (C-1), 62.5 (Glc-6), 28.7 (C-2), 21.6 (C-5), 14.8 (C-6).

## 4. Conclusions

A total of 19 compounds belonging to different chemical classes such as phenylpropanoids, benzoic acid derivatives, flavonoids, coumarins, etc., were isolated and identified for the first time from the genus *Cardiocrinum*. From the results of current study and with the previous reports, it can be assumed that the flavonoids and other phenolic compounds can be considered as main components in the aerial parts including leaves, stems, and seeds, which may serve as the chemotaxonomic markers of these species. Future studies are necessary to isolate and identify chemical constituents from the aerial parts of other two species and comparison of interspecific variation. On the other hand, bioactivity evaluations of the extracts and isolated compounds is necessary to explore the potential of *C. cordatum* for use in pharmaceutical, cosmetic, and functional food industries.

## Data Availability

Samples of isolated compounds are available from authors upon request.
